# Blurred boundaries at the Intergovernmental Panel on Climate Change: the role of integrated assessment models in the science–society contract

**DOI:** 10.1098/rsos.250286

**Published:** 2025-09-10

**Authors:** Simon Robertson

**Affiliations:** ^1^ UNSW Sydney, Sydney, Australia

**Keywords:** IPCC, integrated assessment models, science-policy boundary, science–society contract, equitable and resilient futures

## Abstract

In this article, the broken *science–society contract* contention of Glavovic *et al*. (Glavovic *et al*. 2022 *Clim. Dev*. **14**, 829–833 (doi:10.1080/17565529.2021.2008855)) and their posit of the tragedy of climate change science will be examined in relation to the Intergovernmental Panel on Climate Change’s (IPCC) employment of integrated assessment models (IAMs) in the Sixth Assessment Report (AR6). The article will assess, empirically, Skea *et al*.’s (Skea *et al*. 2021 *WIREs Clim. Change* **12**, 1-11 (doi:10.1002/wcc.727)) IPCC AR6-and-beyond IAM transparency roadmap by appraising the efficacy of the ‘*actions taken*’ for achieving transparency in the AR6. If the IPCC was to earnestly assure the transformation of IAM clarity from its present state of a black-box to that of a glass-box, then its proclaimed mantra of ‘*neutral, policy relevant but not policy prescriptive*’ could be received with high confidence. Until then, the IPCC endangers its objectivity, its integrity and its scientific standing in society owing to the Panel’s non-compliance with the published Principles Governing IPCC Work as to expected transparency standards. Accordingly, the operation of opaque IAMs for purported ‘relevant but not prescriptive’ policy guidance has resulted in the IPCC’s blurring of the science-policy boundary as a consequence of the IPCC-Integrated Assessment Modelling Consortium contingent’s breaching of the science–society contract.

Amory Lovins on the IIASA’s Energy Study: *‘...does its devotion of untold talent and money to an elaborate modeling exercise tell us anything that could not have been shown more persuasively and transparently on a hand calculator; the effect of its computerization is not to produce insight but to conceal biases.*’ [1, p. 32]

## 1. Introduction

In the resolute provocation of Glavovic *et al*. [2, p. 1], the authors opened their topical commentary with the following terse and unreserved declaration: ‘*The science-society contract is broken*’. In addition to the contract being broken, the authors opine that climate change scientists have dramatically failed at bridging the science-policy boundary. Glavovic *et al.* [2] aver that as the science is settled, then the problem is political.

However, perhaps the failure of successive governments and political leaders to halt global warming, as stated by Glavovic *et al*. [2], is attributable to the reality that the science-policy boundary has indeed been successfully bridged, although not in the traditional sense. Rather than the successful bridging having been performed in the conventional manner of boundary interaction, in which the science informs government policy, the politics have begun to inform the science. Illustrative of such ‘policy-science’ boundary interaction is when the European Commission made enquiries of the Netherlands Environmental Assessment Agency (PBL) and the International Institute for Applied Systems Analysis (IIASA) regarding its very specific desire for a new pathway with a lower radiative forcing of 0.3 W m^−2^ than that of the RCP2.9. The Intergovernmental Panel on Climate Change (IPCC) stated that, *‘[d]uring the course of the deliberations of the Panel, the European Commission asked the two European RCP-ready IAMs—IMAGE and MESSAGE—to produce, if possible, 2.6 w/m^2^ scenarios with which they were comfortable. As expected, these scenario [sic] do include a moderate amount of overshooting of the 2.6 w/m^2^ radiative forcing target during the middle of the century, but they are able to get back under the target by the end of the century primarily through the use of bio-energy with carbon capture and sequestration (BECS) [sic], a net negative greenhouse gas emitting technology*’ [3, p. 5]. Despite this technology appearing in ‘feasible’ integrated assessment model (IAM) scenarios promoted by the IPCC, this nascent negative-emissions technology has been criticized for locking in society’s carbon addiction while acting as a ‘*political panacea*’ in that the technology excuses government passivity in terms of failing to enact immediate, deep and rapid mitigation [4]. The existent political economy of mathematical modelling allows epistemic authority to be bought through the utilization of large models that provide the illusion of objectification or depoliticization of political issues [5].

It is then possible that what Glavovic *et al*. [2] refer to as government inaction is actually what Carton [6] has termed as the ‘*imperative of gradualism*’ of IPCC mitigation scenarios. The notion of ‘*imperative of gradualism*’ is that mitigation must be incremental in order to defend the economic benefits of fossil fuel production to the greatest extent possible [6]. Governments’ decisions unequivocally demonstrate that powerful vested interests, such as those of the fossil fuel industry, carry influence much greater than that of the accumulated scientific knowledge on climate change [7]. IAMs’ reductionism of climate change mitigation to a carbon cost, which is then subjected to the equivocal concept of cost-minimization, renders mitigation legible to vested political and economic interests while delimiting the range of seemingly feasible mitigation options [6]. Scrutiny of IAMs must extend to include the political work that the IPCC scenarios perform as well as the logical basis from which these modelled outcomes have emerged [6].

Adopting such alternative framing, this focused article will examine Glavovic *et al*.’s broken *science–society contract* contention and their posit of the tragedy of climate change science in relation to the IPCC’s employment of IAMs in the Sixth Assessment Report (AR6) to inform policymakers on climate change mitigation strategies. Fundamentally, this contract anticipates that the outcome of publicly funded scientific research will be beneficial to society. The authors’ assertion of government inaction on climate change mitigation as a failure index of the science–society contract [2] is relevant to the IPCC’s furtherance of IAMs for policy guidance. This is particularly so when, according to Working Group III Mitigation (WGIII) AR6 co-chair Skea’s calculations, IAMs constitute up to 6% of the report contents, approximately 25% of the summary for policymakers and the best part of the press coverage (see [8]).

Having received trenchant criticism concerning the lack of transparency of IAMs in the Fifth Assessment Report, the IPCC provided much attention to addressing this issue in the preparation of the AR6. The published scoping document for the AR6 acknowledges that ‘*[t]he role, the construction and the transparency of the assumptions underlying these models has since been challenged*’ [9, p. 48]. In response, the IPCC gave a commitment for the AR6 to ‘...*improving the transparency of assumptions and approaches associated with scenarios and modelling*’ [9, p. 54]. This commitment was enumerated in Skea *et al*. [10].

Owing to the critical role of the IPCC as a science-policy boundary facilitator, this article will assess, empirically, Skea *et al*.’s [10] IPCC AR6-and-beyond IAM transparency roadmap by appraising the efficacy of the ‘*actions taken*’. These ‘*actions taken*’, according to Skea *et al*. [10, p. 5], were steps ‘*…taken to enhance the transparency of modeling and scenarios in the WG III AR6 report*’.

Skea *et al*. [10] comprised the WGIII co-chairs for the AR6, with both co-chairs having roles at the Integrated Assessment Modelling Consortium (IAMC); the remaining authors were from the IPCC WGIII Technical Support Unit, with one of these authors also affiliated with the IAMC. Although Skea *et al*. [10] declare explicitly their IPCC association as a ‘conflict of interest’, the authors fail to declare additional conflicts of interest in that Imperial College London (Skea and Al Khourdajie), Ahmedabad University (Shukla) and the Electric Power Research Institute (McCollum) were members of the IAMC (see [11]), as well as three of the four authors failing to declare their respective appointed roles at the IAMC. As such, Skea *et al*. [10] was produced and published as an IPCC–IAMC joint communiqué.

Given that the IPCC has been well aware of the IAM transparency problem since first being prophetically identified more than a quarter of a century ago by Risbey *et al*. [12], the Panel’s AR6-and-beyond IAM transparency roadmap, as presented in Skea *et al.* [10], is equally salient to the scientific community, government and the general public. The efficacy of the IPCC’s IAM transparency roadmap is paramount, especially when Shapiro *et al*. [13] determined that transparency was lacking at the IPCC, with such an impact upon the promotion of trust with the public, with the scientific community and with governments.

Science occupies a distinguished position from that of other policy inputs by reason of its relative objectivity, with this being attributable to its formal processes of data collection and analysis aimed to minimize bias [14]. Traditionally, the understanding of the dynamics across the science-policy boundary has been one in which science informs policy. However, science-policy boundary operations between scientists/researchers and users of scientific information, such as policymakers, occur in various modalities: (i) ‘science-push’ mode—the pursuit of knowledge is the catalyst for scientific production and the applicability of this knowledge as a solution is determined by the problem, (ii) ‘demand pull’ mode—science is commissioned or solicited by stakeholders in response to the desire for a solution to a problem, or (iii) a ‘co-production’ mode—an iterative exchange combining both push and pull between scientists and stakeholders, respectively [15]. An integral question to be asked of all boundary modes is: who is driving the agenda for what knowledge is produced? [15]. Such an enquiry reveals the mode in operation. In the case of the operations between integrated assessment modellers and policymakers, it could be argued that ‘demand pull’ is the dominant mode between these two actors. This is best represented by policymakers requesting from the integrated assessment modellers mitigation pathways that were Paris Agreement compatible (see [3,16]). While this mode of operation is not deleterious *per se*, it does present a hazard when such subsequent information is, to all intents and purposes, scientifically generated in the absence of transparency. With the ‘demand pull’ mode potentially generating information that is scientifically infeasible or lacking scientific robustness [15], transparency becomes paramount, particularly when such science engages with the science–society contract. If this information is produced in an opaque manner, then what is presented may represent political fiction rather than scientific fact. As the ‘demand pull’ mode relates highly to the production of non-transparent IAM products, this then becomes highly problematic for the IPCC with its purported apolitical position. Science that is performed in response to ‘demand pull’ boundary interactions and done so without transparency endangers the blurring of the science-policy boundary in that the mode appears purely scientific (i.e. science-pull), free of external influence from non-scientific interests. Consequently, the IPCC’s foregrounding of non-transparent IAMs for purported ‘relevant but not prescriptive’ policy guidance risks the blurring of the science-policy boundary as a result of the IPCC–IAMC contingent’s potential non-compliance with the science–society contract. Science that is produced in response to a ‘demand-pull’ science-policy boundary mode is distinctly probable on balance to involve vested political and economic interests, and thus transparency is vitally important.

Moreover, the inescapable involvement of values and judgement is associated with the economic inner-workings of IAMs. IAMs are primarily concerned with the economics of technological change and the resultant emissions response. Distinct from models grounded in well-established physical laws, such as general circulation models, economic models such as IAMs with their ‘*shakier*’ foundations are deserving of ‘*close and critical scrutiny*’ owing to their melding of value judgement with descriptive analysis [17]. Indeed, the IPCC WGIII, in Annex I (p.14) and Annex III (p.56) Final Government Distribution (FGD) AR6, acknowledges the value judgements intrinsic to the economics of IAMs in relation to the debate concerning the choice of the discount rate and the arguable lack of sensitivity analysis as to how such a rate affects the modelled outcomes [18]. This overarching economic bias in IAMs potentially exposes the opaque modelling endeavour to outwardly scientific influence, which in turn makes the science-policy boundary more susceptible to being blurred, that is to say, the science-policy boundary mode.

Skea *et al*. [10] argue that IAMs have been subjected to a high level of scrutiny given their prominent role in climate policy debates. However, the accuracy of such a statement has been disputed (see [19]). Fundamentally, if model code is not open-source and/or input data are not publicly available, then it is unreasonable to claim that IAMs and their products have been subjected to a high level of scrutiny. The lack of scrutiny resulting from an absence of transparency is problematic for inherently political IAMs, which assume primacy in the development of globally coordinated mitigation strategies [19]. Purvis [19] asserts that the majority of IAMs have managed to avoid entirely the level of critique other well-documented models have received. This indicates that for models, which sit within and without hegemonic tradition, the playing field is not level [19]. This lack of a level playing field granted to IAMs is reflected in *Nature Climate Change*’s journal editorial, where the editors maintain that experts can only probe the robustness and meaningfulness of their outputs when the underlying assumptions as well as model inputs are made explicit [20]. In public science, unfortunately, institutional cultures of self-interest within powerful organizations foster a type of deplorable secrecy where scientific facts that the public is entitled to know are purposefully concealed and knowingly suppressed in order to preserve the economic or political position [21]. Interests and power structure opacity in science undermine desired environmental protections, which sustains the unproductive science status quo [22].

The article is organized as follows. Section 2 is concerned with the determination of the efficacy of Skea *et al*.’s [10] IPCC AR6-and-beyond IAM transparency roadmap. This section comprises two principal subsections. Section 2.1 identifies the transparency indicators. Section 2.2 evaluates the transparency initiatives undertaken by the IAM community and by the IPCC with respect to the nominated indicators, their cross-interactions and consequent interdependence. The structure of this subsection reflects the cross-interactions and consequent interdependence between indicators and remedial actions. Section 3 examines the ramifications of non-transparent IAM scenarios on climate change policy, their discordancy with The United Nations Educational, Scientific and Cultural Organization’s (UNESCO) advocacy for Open Science and their consequential impact on the fulfilment of the United Nations (UN) Sustainable Development Goal (SDG) 13 of ‘Climate Action’. Section 4 provides an overall judgement of the article’s findings with respect to policy implications.

## 2. The questionable efficacy of integrated assessment model transparency initiatives

### 2.1. Indicators of transparency according to the Intergovernmental Panel on Climate Change AR6-and-beyond integrated assessment model transparency roadmap

In Skea *et al*. [10, p. 3], the authors pose the principal question with respect to IAMs of ‘*What does transparency mean*?’, further refining this interrogation in terms of ‘…*transparency for whom*’. After posing these questions, Skea *et al*. [10] then enumerate the following aspects of the modelling endeavour as indicators of transparency: *model structure*, *input assumptions*, *model equations*, *model code* and *modelling strategies*.

Skea *et al*.’s nominated indicators of *model structure*, *model equations* and *model code* are heavily interdependent in terms of demonstrating model transparency. In other words, the model code is the programming linguistic representation of the model equations, which are, in turn, a mathematical expression of the model structure. Therefore, if the aforementioned transparency indicators are not entirely forthcoming, then full and proper transparency of the model is unattainable. The indicator of *modelling strategies*, as asserted by Skea *et al*. [10], interacts with input assumptions such as the discount rate, with the authors acknowledging the dearth of appropriate documentation, as well as citing Bistline *et al*.’s [23] call for the structural assumptions and value-laden assumptions to be made explicit. However, Skea *et al*. [10] fail to establish how this indicator, characterized by constrained greenhouse gas concentrations or global warming levels, aids in transparency. The remaining indicator of *input assumptions* will be discussed in detail below in relation to the IPCC’s ‘*actions taken during the AR6*’ initiatives of scenarios database and data archival and curation, in addition to the modelling community’s ‘*actions taken*’ of scientific papers. It is critical to note the distinct indicators of model code and input assumptions as stipulated in Skea *et al*. [10]; these indicators are independent and are not interchangeable (see [24], but cf. [25]), as each indicator captures a separate aspect of transparency related to the modelling endeavour. This will be discussed in detail in §2.2.3.

Having established their transparency indicators, the authors then document ‘*actions taken during the AR6*’ by the IPCC in an attempt to demonstrate a conscious and concerted effort towards transparency, as well as detailing ‘*actions taken*’ by the IAM community. With that said, and despite the designated transparency indicators of *model code* and *input assumptions*, Skea *et al*.’s [10] fundamental negligence in making the critical distinction between model transparency and scenario transparency is concerning. Another critical distinction that Skea *et al*. [10] neglect to make is the related process of applying their nominated transparency indicators. In order to function as intended, the authors’ transparency indicators must be applied as a suite. Therefore, until all indicators indicate transparency for both model and scenario, full transparency cannot, and indeed must not, be claimed. An examination of such aspects is to be discussed hereinafter.

### 2.2. Transparency initiatives undertaken by the integrated assessment model community and the Intergovernmental Panel on Climate Change

#### 2.2.1. Actions taken by the integrated assessment model community

Explicitly linked to these cognate model transparency indicators are the alleged actions, as noted by Skea *et al*. [10], which have been undertaken by the IAM community of *documentation*, *open-source models* and *scientific papers*. Mindful of the distinction between model transparency and scenario transparency, as overlooked by Skea *et al*. [10], the production of appropriate documentation must be classified into corresponding publications for the model and for the scenario. While there is a gross inadequacy of appropriately detailed scenario documentation, as will be discussed and as curiously conceded by Skea *et al*. [26], model documentation, on the other hand, is, in general, sufficient in detail. Complementing the production of model documentation is the open publishing of model code, which is also an essential component towards openness and transparency. However, as previously stated, the transparency end result in such an instance is limited only to the model and does not extend to the associated scenario runs. It should be recognized that the open-source publishing of model code is not a universal practice across the members of the IAMC (e.g. table 1 in [25]). Skea *et al*. [10, p. 7] rightly note the fact that the mastery of open-source model code by a non-native user in terms of comprehension and execution requires domain knowledge, an understanding of specific research methods and scientific computing skills. Regardless of these epistemic barriers, such potential knowledge deficits must not be used as a show cause for remaining closed with code or data. Unfortunately, such an approach is present in the tendentious article of Keppo *et al*. [25, p. 13]. Gatekeeping on the basis of limiting the potential for the misuse or mitigating the risk of misunderstanding by non-IAM scholars is without *bona fide* justification. After all, as a structured medium for conveying methodological concepts, ‘*…programs must be written for people to read, and only incidentally for machines to execute*’ [27, p. xxii].

Furthermore, while the ‘*action taken*’ of *model intercomparison projects* (MIPs) creates a potential arena for testing the sensitivity of nominated assumptions for a like scenario across differing IAMs, MIPs are ineffective in terms of scenario transparency and specificity. In fact, the IPCC conceded in its Second Assessment Report (SAR) [28, p. 392] with respect to last century’s IAMs, *‘[t]he larger a model, the less transparent it is, and the harder it is for analysts and policymakers to interpret its results. The sheer size of the model renders full sensitivity analyses impossible, and it becomes more difficult for the modellers themselves to oversee what is happening*’. In summary, and perhaps somewhat ironically, the larger the IAM, the more important transparency becomes, which is hindered by the consequential fact that the larger the IAM, the less transparent it becomes. This concession by the IPCC in its SAR only further serves to emphasize the essentiality of IAM scenario input data disclosure as a practicable approach to IAM scenario transparency. Skea *et al*.’s [10] final alleged demonstrated ‘*action taken*’ performed by the IAM community of *scientific papers* will be discussed below in the context of their nominated transparency indicator of *input assumptions*.

#### 2.2.2. Actions taken by the Intergovernmental Panel on Climate Change

In addition to the purported ‘*actions taken*’ executed by the IAM community, Skea *et al*. [10] enumerate the IPCC’s ‘*actions taken during the AR6*’ consisting of the *scope of the WGIII AR6 report*, *author selection*, *scenarios database*, *data archival and curation* and *engagement with a wider range of stakeholders*.

Similarly to that of the purported purpose of *model documentation* is the accompanying annex to chapter 3 of the WGIII AR6, as listed under the ‘*action taken*’ of *scope of the WGIII AR6 report*. Skea *et al*. [10] highlight that the report will provide an annex dedicated to scenarios and modelling methodologies, thereby demonstrating an ‘*action taken*’ for aiding in IAM and associated scenario transparency. The said annex, that is to say Annex III, is of limited use when it comes to unpacking the nuances of the AR6 ensemble of IAM mitigation scenarios for the policymaker and, as such, is limited in terms of demonstrating scenario transparency. Annex III fails to ‘*…state the exact policy assumptions upon which the scenarios were developed’* [29, p. 1].^1^ Unlike that of the general adequacy of model documentation, scenario documentation, which of necessity should accompany the full disclosure of Skea *et al*.’s [10] transparency indicator of *input assumptions,* is effectively non-existent. This will be discussed hereinafter.

The authors [10, p. 5] then proceed to acknowledge that for the AR6, the WGIII report ‘…*divides the future into two timeframes*’ with chapter 3 having a time horizon end point of year 2100, while chapter 4 addresses mitigation and development pathways in the near- to mid-term. Other than enumerating this alleged transparency ‘*action taken*’, Skea *et al*. fail to explain how this results in improved IAM transparency, particularly when, according to the authors’ [10, p. 5], ‘*[t]he latter chapter covers models other than those that might be classified as IAMs*’. Paradoxically, and contrary to the unfounded assertions of Skea *et al*. [10], the addition of chapter 4 does not even remotely address the issue of opaque global IAMs. This then leaves policymakers without awareness of national mitigation specificity. Confirmatory of the incompatibility between chapter 3 and chapter 4, and thereby a failure of this action taken in improving global IAM scenario transparency, is the following statement taken from the approved chapter 4 FGD [18, chapter 4, p. 8] where it explicitly states that, *‘[t]he national scale selected in this Chapter requires attention as national mitigation pathways cannot be linked directly to global mitigation goals (see Box 4.2*)’. As a matter of fact, the revision for chapter 4 of the FGD in the now corresponding Box 4.2 only confirms and highlights this linking problem where the authors of chapter 4 [18, p. 32] cite Robertson [30] and Rogelj *et al*. [31] noting that, ‘*[d]irect links between an individual actor*’*s mitigation efforts in the near-term and global temperature goals in the long-term cannot be inferred; making direct links requires clear distinctions of spatial and temporal scales’*. A fixation on global trends has the potential to obscure important changes at more granular levels [32].

Interestingly, and in the same year of publication of the IPCC WGIII’s contribution to the AR6, it has indeed been verified by a group of IAM practitioners that global scenarios produced by global IAMs in their current form are of limited use in informing national or regional mitigation strategies (see [33]). Köberle *et al*.’s [33] findings have effectively rendered the AR6 global IAM mitigation scenario ensemble to be inadequate for use by policymakers at the national level and/or policymakers at the regional level. Consequently, this inconvenient truth must also hold true for all IPCC antecedent reports that have relied upon global IAMs for national and/or regional climate change mitigation policy guidance.

Following on is the ‘*action taken*’ of *author selection*. For Skea *et al*. [10] to assert author disciplinary diversity for chapter 3 as an ‘*action taken*’ to bring about IAM transparency is without foundation, as it does not provide absolute assurance. The authors ought to know that author disciplinary diversity, *per se*, is an inappropriate action for demonstrating IAM-related transparency. Skea *et al*. [10] should be aware that it is, in fact, author participation, with this being reflected in the disciplinary diversity of an activity’s intellectual output, that may act as a suitable measure of transparency in terms of explicitly addressing bias types, such as academic bias and/or confirmation bias.

In an effort to provide unprejudiced validation as to the legitimacy of my unfavourable estimation of this particular ‘*action taken*’, it is of interest to survey the publicly available, independent review comments submitted by IPCC WGIII vice-chair Diana Ürge-Vorsatz for the First Order Draft of chapter 3. Ürge-Vorsatz’s comments document the unfortunate state of affairs in the chapter, especially the underuse of the chapter’s disciplinary diverse lead author group composition with this being reflected in the overall IAM-centric content of the chapter. This exclusionary and exploitative behaviour by the IPCC–IAMC contingent is all too reminiscent of the interviews from participating scientists reflecting upon their author experience of the IPCC Fifth Assessment Report (AR5) (see [34]). With the identical problem regarding the dominance and uncritical assessment of IAMs having seemingly occurred in chapter 6 of the AR5, which resulted in the departure of non-IAM lead author David Keith, it would appear that the IPCC–IAMC contingent suffers from pronounced co-institutional ossification. Suggestive of this particular ‘*action taken*’ being tokenistic is perhaps affirmed by the IPCC Bureau’s parochial decision to appoint only IAM scholars to the roles of coordinating lead authors for chapter 3, with both of these individuals being senior members of the IAMC. These intentional appointments to the proclaimed disciplinary diverse chapter 3 of the WGIII AR6, not remotely indicative of the much-touted author diversity as promoted in Skea *et al*. [10], created an instantaneous bias within the chapter. Such appointments and the resultant bias contravene paragraph 12 of the IPCC’s Conflict of Interest Policy and the rationale for compliance as captured in paragraph 2 of the policy.^2^

#### 2.2.3. Actions taken by the integrated assessment model community and the Intergovernmental Panel on Climate Change regarding the *input assumptions* transparency indicator

Before commencing this concluding part of the analysis of Skea *et al*. [10], it is necessary to pay particular attention to the following assertion of the authors [10, p. 2]*—‘Progress can be made on substantive issues only if debate is well-informed. Transparency about methods and assumptions is an absolute prerequisite in this respect*’.^3^ This statement from Skea *et al*. [10] undergirds the proceeding detailed discussion of their transparency indicator for *input assumptions* and related ‘*actions taken*’ of *scenario database*, *as well as data archival and curation* by the IPCC for the AR6 and *scientific papers* by the IAM community.

Skea *et al*. [10] declare the ‘*action taken*’ of *scenarios database* and the associated action of *data archival and curation* as IPCC devised IAM transparency initiatives for the AR6. These ‘*actions taken*’ by the IPCC and the degree to which each offers to IAM scenario transparency are profoundly influenced by the authors’ transparency indicator of *input assumptions*, notwithstanding the authors on the one hand flagging this indicator’s importance but on the other hand dismissing its applicability.

In the case of the *scenarios database*, this extant transparency initiative from the IPCC has been assessed as being deficient when demonstrating IAM scenario transparency on account of the omission of critical scenario input data. The failure of previous IPCC scenario databases identified by Robertson [30] was recently conceded in Pedersen *et al*. [36]. Pedersen *et al*.’s concession only reaffirms Robertson’s assessment that the claim espoused in Huppmann *et al*. [37] concerning the rationale for the IAMC 1.5°C Scenario Explorer database was sophistic. This is especially so when the authors erroneously evoke the FAIR data principles as a means of bolstering the purported effectiveness of the IAM scenarios database transparency initiative. Such an unfavourable assessment of the IAMC 1.5°C Scenario Explorer database disappointingly holds true for the IAMC AR6 scenarios database. In an effort to counter this shortcoming, Skea *et al*. [10, p. 6] announce that for the AR6, ‘…*the call for scenarios (IIASA, 2021a) was extended to include a wider range of technical parameters, including the assumed capital and operating costs of individual technologies, and service demand levels (e.g., floorspace, passenger kilometers)*’*.* However, the Panel’s call is without merit, particularly when scenario input datasets are not fully disclosed and not mandated as a prerequisite for submission to the IPCC scenario assessment process. Similar to Huppmann *et al*.’s [37] assertion for the IPCC Special Report on 1.5 degrees (SR1.5), Kikstra *et al*. [38, p. 9081] assert that for the WGIII AR6, the ‘*increased attention*’ to ‘*documenting the core assumptions and characteristics of IAMs*’ was ‘*to facilitate their interpretation and reproducibility*’. Facilitation in the interpretation of IAM scenarios? Conceivably. Facilitation in the reproducibility of IAM scenarios? Most definitely not. Robertson [30, p. 6] opines that, ‘…*the AR6 scenario database must, of necessity, contain not only model output but also model input…detailing the underlying assumptions for each scenario assessed. Failure to do so will be expressly contrary to the IPCC’s AR6 published commitment’.* The IPCC’s failure on this matter will be discussed further with respect to Skea *et al*.’s [10] transparency indicator of *input assumptions*.

The IPCC’s ‘*action taken*’ for IAM transparency during the AR6 of *data archival and curation* draws its effectiveness from the preceding ‘*action taken*’ of *scenarios database*, as the process of archival and curation is simply an anticipated act of IPCC housekeeping. It is nonsensical for the IPCC to establish a Task Group on Data Support for Climate Change Assessments and to then subsequently devise ‘…*guidelines aimed at enhancing the accessibility and transparency of data and scenarios underlying IPCC reports’,* as stated in Skea et al. [10, p. 6], when its principal objective is to only provide ‘…*full documentation, including data, metadata and information on the data and scripts used to produce…figures, tables and exhibits’* [10, p. 4]. As was the case with the publication of the IAMC 1.5°C Scenario Explorer database, the IAMC AR6 Scenario Explorer database that accompanies the IPCC WGIII’s contribution to the AR6 has the potential to be interpreted as being a disingenuous attempt at transparency through the spurious use of the scientific notion of reproducibility. Reproducibility, in the instance of the AR6, is in fact once again, as it was with the SR1.5, limited to the ‘data’ ‘…*used to produce…figures, tables and exhibits’* (e.g. [10, p. 6]) and not that of the data fed into the suite of IAMs used to generate the ensemble of global mitigation scenarios including baseline scenarios. An IPCC IAM scenarios database without input data is a misnomer [30].

Central to these IPCC’s ‘*actions taken during the AR6*’ of *data archival and curation* and *scenarios database*, as well as the modelling community’s ‘*action taken*’ of *scientific papers*, is Skea *et al*.’s [10] transparency indicator of *input assumptions* that can be understood as being a representation of IAM scenario input data.

Skea *et al*. [10, p. 4] recognize that ‘*IAMs require large arrays of input assumptions*’. The authors [ 10, p. 4] then assert, without any form of substantiation, that, ‘*[f]ull transparency would require published documentation of all assumptions, but this is patchy in practice*’. Since neither the IPCC nor the IAMC has ever ‘*published documentation of all assumptions’*, what ratiocination have the authors exercised in arriving at the unsupported conclusion that such an endeavour ‘...*is patchy in practice’*? This counterfactual assertion by Skea *et al*. [10] does not remotely assist in IAM scenario transparency due to its pernicious, equivocatory effect and only serves to rationalize the IPCC’s continuing inaction on IAM scenario input data disclosure. Moreover, if this is the case for unpublished input assumptions, then, on the authority of the authors, ‘*full transparency*’ of IAMs and their scenarios can never be attained, regardless of the IPCC–IAMC contingent’s well-publicized and much-heralded actions taken. As with any scientific undertaking, the conscious and deliberate withholding of data is not remotely conducive to transparency.

During a 2021 interview with Michael Liebreich, IPCC WGIII co-chair Skea stated the following with regards to the call for scenario submissions to the IPCC AR6 database*—‘And we also think that some people may have worried about confidentiality, because some of the issues do involve making assumptions, exposing the assumptions you*’*ve made about things like prices, the costs of technologies, which some people might find quite sensitive*’ [39]. *Ipso facto,* this reasoning of Skea must logically extend to the assessment of scenario outputs, in that, the IAM scenario runs must also embody a commensurate degree of sensitivity owing to the innate sensibility of input assumptions as determined by the modellers. If the scenario input data are not fully declared due to concerns over confidentiality and/or sensitivity, as asserted by Skea, then the resultant scenario output is patently unfit to be used by governments worldwide in formulating attendant climate change mitigation policy. Similar to Skea’s sentiment expressed to Liebreich, and somewhat perplexingly given the publication year is identical to that of Skea *et al*. [10], although relatively contradictory, Skea *et al*. [26, p. 17] concede that,*‘…it may be relevant to assess similarities and differences in terms of model input assumptions…This approach is more challenging because of the lack of published information and possible sensitivities about disclosure*’.

Statements in the vein of ‘…*making code and data publicly available is valuable…but few people know how to run and critique a model of this kind*’ as operationalized by Keppo *et al*. [25, p. 14] are bromidic. The matter of whether or when data are to be disclosed is not a decision to be determined by the IAM community, nor is it a matter for the IPCC. The Organisation for Economic Co-operation and Development (OECD) reasons that when research data are generated through public funding, then access should be made available [40], which, most certainly, holds true for the IAM scenario activities that are so heavily reliant upon government funding arrangements [30]. Purported pseudo-proprietary data that are generated by publicly funded research activities, as could be argued to be an accurate assessment of the iniquitous present state of IAM scenario input data, are in every respect incompatible with both UNESCO’s Open Science credo and the OECD’s principles and guidelines for the access to research data from public funding. Moreover, and despite the IPCC’s published commitment for IAM scenario transparency in the AR6 Scoping Document (see [41]), the Panel’s continued inaction as to IAM scenario input data transparency has the potential to be interpreted as being the IPCC’s disingenuous commitment to transparency, its apathetic adoption of the InterAcademy Council’s (IAC) report recommendations (see [13]), and its contempt for the accepted convention in terms of adhering to expected rigorous scientific standards as codified in the Principles Governing IPCC Work. In order to forestall the possibility of conjecture, it is important to remember Skea *et al*.’s failure to differentiate between model transparency and scenario transparency and the authors’ remark concerning ‘*domain knowledge*’. However, I trust that Skea *et al*. [10], as well as the likes of Keppo *et al*. [25], are not mistakenly conflating model ‘*domain knowledge*’ with the knowledge required to comprehend scenario input data, given that model ‘*domain knowledge*’ is not a prerequisite when assessing the validity of model scenario input data. As previously discussed, Skea *et al*.’s [10] indicator *model code* is distinct from their additional indicator of *input assumptions*; in that, the data are inputted into the model, which, through processing via the code, outputs the information. Alarmingly, Keppo *et al*.’s [25, pp. 3−4] table 1, which details the heterogeneity of global IAMs, confirms that for the authors’ declared category of ‘*Model transparency*’, the declared indicator of ‘I’, which denotes ‘*input data publicly available*’, was absent for all nine major IAMs reviewed, and that for five out of the nine models, ‘*open source code*’ denoted by indicator ‘C’ was also unavailable. Indeed, as specified in the authoritative Guidelines for Transparency and Openness Promotion (TOP) in Journal Policies and Practices, transparency guidelines for data and for code are ‘*conceptually distinct*’, with each constituting a seperate transparency standard across all stringency levels covered by the TOP Guidelines for open practices and in the promotion of Open Science [24, p. 3].

In an exercise of *reductio ad absurdum*, the present position is that some IAM practitioners are more than willing to open publish the model code which apparently requires ‘*domain knowledge*’ in order to comprehend its logic, but all practitioners are unwilling to open publish scenario input data on the fallacious grounds of requiring relevant ‘*domain knowledge*’ for comprehension. This *non sequitur* of having open-source models together with published scenario output, while at the same instant scenario input data remain under lock and key, is not conducive to the transparency outlined in IPCC [9] and as advocated in Skea *et al*. [10]. Remaining cognisant of the contradistinction between soundness and validity, the accepted principle of the canonical acronym GIGO states: GARBAGE IN-GARBAGE OUT. The full disclosure of output datasets must coincide with the full disclosure of input datasets so as to improve the transparency of IAMs [42]. When the modelling process and peer review system encourage the building and publication of models where critical factors are omitted, then there is something ‘*fundamentally wrong*’ [43, p. 519].

As a means of reasserting the IPCC’s enterprising preparation for the AR6 cycle, Skea *et al*. [10] set forth the proposed recommendations regarding the presentation of IAMs in the AR6 (see [9]). It is notable to observe that Shukla, as Break-Out-Group (BOG) facilitator on the topic of ‘Model Intercomparison Projects and marker scenarios’, advocated that [9, p. 11], ‘*Data transparency should be strongly encouraged. Open access data and open source modelling tools should be encouraged across all modelling communities*’. Moreover, BOG facilitator, Øyvind Christophersen, stated [9. p. 12] that, ‘*Be open with data. This helps demonstrate transparency and builds trust*’. Such a statement would suggest, as expected, that being closed with data must result in the hindrance of transparency and in the erosion of trust.

As previously referred to in this subsection, Skea *et al*. [10, p. 2] opine that, *‘Progress can be made on substantive issues only if debate is well-informed. Transparency about methods and assumptions is an absolute prerequisite in this respect. Transparency…facilitates debate and provides a clearer evidence base for policymakers*’. Owing to the fact that the IAM scenarios, which appear in the AR6, fail the authors’ ‘*absolute prerequisite*’ of ‘*transparency about methods and assumptions’* so as to enlighten a ‘*well-informed debate*’, then the policymakers must subsequently be, according to Skea *et al*.’s declared logic, without ‘*a clearer evidence base*’ from which to work from when devising policy. This statement of the authors is not at all assisted by Skea’s inimical attitude of input data non-disclosure on the grounds of third-party confidentiality or sensitivity, as expressed by Skea to Liebreich. As a senior IPCC representative for the AR6, Skea’s implied assent of input data non-disclosure owing to confidentiality and/or sensitivity is certainly not conducive to the realization of transparency as promulgated by the IPCC, nor in the performance of Open Science. Notably, the IAC found that the IPCC’s lack of access provision to data for critics and enquirers had contributed to controversies surrounding the Panel [13]. Even with privately funded research, there is a compelling case for greater openness of data and information when such research has the potential to impact the public [44].

The final transparency ‘*action taken*’ by the IAM community of *scientific papers*, as promoted in Skea *et al*. [10], is limited in demonstrating IAM scenario transparency, particularly with the authors’ unscientific dismissal of their *input assumptions* transparency indicator. Unfortunately, varying practices for IAM scenario reporting have led to difficulties for policy analysts in inferring meaning when comparing results [32]. The significance of addressing this and associated challenges has led to calls for improved disclosure and greater transparency [32]. So as to overcome these difficulties that confront policy analysts, decomposition tools may be applied, which allows for the sanity checking of scenario design such as the reliance upon heroic assumptions often made in scenarios (e.g. bio-energy with carbon capture and storage (BECCS)), the promotion of transparency thereby facilitating debate on key issues and uncertainties in mitigation scenarios, and by assisting in the valid comparison of results generated by multiple models [32]. Nevertheless, the quality of results that decomposition tools produce is only as good as the quality of the input data [45]. Scenario intercomparability is only feasible if each and every input assumption is made transparent [46].

#### 2.2.4. Actions taken for external stakeholder engagement

While the final transparency ‘*action taken*’ by the IAM community was *scientific papers*, Skea *et al*. [10] advance the final transparency ‘*actions taken during the AR6*’ by the IPCC of *engagement with a wider range of stakeholders*. Skea *et al.*’s ‘*action taken*’ by the IPCC of *engagement with a wider range of stakeholders* for the AR6 as a transparency initiative is scant in detail and dubious in its claims of efficacy. The authors’ detailing of the all-inclusive AR6 webinars instigated by the IPCC as an example is without substantiation when it comes to specifically improving IAM scenario transparency. Having attended such webinars as a WGIII chapter 3 lead author, I am bemused as to how such a post-production initiative aided in IAM scenario transparency. However, unlike the IPCC outreach webinars, engagement in the modelling process must not be bookended but must be on a continual basis from inception to completion, an open, transparent and reflexive process. However, the effectiveness of this ‘*action taken*’ is dependent upon the effectiveness of all other transparency initiatives detailed in Skea *et al*. [10] if meaningful and equitable stakeholder engagement is to have any prospect. The late Stephen Schneider astutely remarked last century that, ‘…*vigorous outreach programs…demand transparency and accessibility, and that all values and assumptions that might be hidden in the analytic complexity of IAMs are purposefully made explicit to users…To do less is to make IAMs at best irrelevant to policy-makers, and at worst, misleading*’ [47, p. 246].

Under this IPCC ‘*action taken*’ for the AR6, the authors acknowledge that the IAM scenarios presented by the IPCC are both cited and used by governments, industry, the financial sector and non-governmental organizations. This acknowledgement of end-user diversity by the authors is best illustrated by the IAMC’s decision to recently form the new Scientific Working Group on Financial Analysis. As announced by the consortium, this new IAMC Scientific Working Group recognized the wide range of potential users of scenarios, the very scenarios as reported by the IPCC, for climate-related financial analysis. This targeted application of IAMs rather dangerously tempts fate in morphing already opaque, biased, nominal exploratory scenarios into performative pathways and only calls attention to the most urgent need for full transparency of IAM scenarios. Keppo *et al*.’s [25, p. 14] placation of, ‘…*IAM teams work closely with governments but are not directly controlled by them’*, is somewhat undermined by the authors’ concession in the following paragraph that, ‘...*those in positions of political power influence which possibilities are mapped by the modellers…’*. Analyses produced by IAMs must be guarded against the danger of *‘falling prey to political expediency*’ [12, p. 370]. Highlighting the politicized roles of technocrats, it has been asserted that a triangular relationship exists between the finance sector, the technocracy and elected politicians [48]. This risks seemingly independent technocrats and politicians becoming ‘*hostages*’ of the financial sector [48]. Therefore, the condition resulting from such triangular relations is not a technical issue but rather a democratic issue concerning the control of our economic and social futures in terms of crisis prevention [48]. These forms of institutional arrangements are inconsistent with the existing science–society contract [49]. As such, the formation of the new IAMC Scientific Working Group on Financial Analysis further jeopardizes the purported ‘*neutral, policy-relevant but not policy-prescriptive*’ reports as prepared by the IPCC owing to the likelihood of influence from vested interests in IAM mitigation scenarios, the very same opaque scenarios that feature in the Panel’s Assessment Reports. The corollary of such influence is the potential for the IPCC’s advancement of solutions that are ‘*policy-prescriptive but not policy-relevant*’ [30, p.6]. The differentiation of an IAM as a ‘*truth machine*’ from that of a ‘*heuristic tool*’ is indistinct ([12]; see also [50]).

## 3. Ramifications of non-transparent integrated assessment model scenarios on climate change policy

The ramifications of non-transparent IAM scenarios on climate change policy are manifold. With respect to the allocation of the rapidly diminishing carbon budget, it has been posited that the global IAM scenarios that feature in the AR6 ignore the global North’s carbon dioxide emissions legacy while disregarding the development goals of the global South in terms of future energy needs [51]. Suggestive of a latent bias towards coloniality in the scenario narrative, which is concealed by the process of aggregation to the global level, the climate change burden is being disproportionately placed upon those from the global South, while those from the global North benefit from an increase in energy consumption unobstructed by fossil fuel constraints [51]. With each technological promise, such as those promoted in IAMs, ‘*moral corruption*’ is exacerbated, resulting in ‘*current elites*’ being enabled to pursue self-serving pathways while also allowing them to pass off risk onto vulnerable people in the global South and posterity [52]. IAMs function to reproduce inequality while enabling the reproduction of types of economy that are incontrovertibly unsustainable [53]. The fact that IAM research is distinctly presided over by those in the global North, compounded by the subjectivity of model assumptions, elicits the compelling question as to who is involved in the modelling practice and, more importantly, who is not [54].

If modelled futures produced by IAMs and their accompanying narratives imply and replicate the interests, values and needs of a select group at the expense of others in terms of prioritization, then the epistemic, institutional and ideological attributes must be scrutinized as a matter of justice [55]. IAMs involve substantive bias across three different levels of justice, namely intertemporal, global and social [56]. The practice of good modelling is to be considered as a social activity and thus cannot be performed by modellers alone [57], especially when it concerns the determination of socially equitable and ecologically resilient futures. A partial redress to such development risk as detailed above is the adoption of Open Science practices. As recognized by UNESCO [58], Open Science can act as an effective tool in reducing inequalities between and within countries.

The IPCC’s prolonged failure to open the black-box of IAMs is in every respect incongruent with UNESCO’s definition of Open Science (see [59]). Open Science is imperative in accelerating the attainment of the UN SDGs [60] and, by default, is crucial to SDG Goal 13 of ‘Climate Action’. However, attaining this goal has been made more complicated on account of its pursuit being overwhelmingly (mis)guided by the IPCC’s foregrounding of opaque global IAM mitigation scenarios, the very antithesis of Open Science. Failure to publish data is in total contravention of UNESCO’s Recommendations on Open Science, with its commitment to ‘*Open research data*’ so as to encourage, *inter alia*, ‘*transparency, scrutiny, critique and reproducibility*’ [58, p.18].

Scenario transparency is of critical importance for policymakers. Scenario input data are, in essence, proxies for policy that represent technological and/or behavioural change within the IAM world and which are envisaged to eventually become implemented, in one way or another, in the real world over the course of this century. As a matter of fact, the IPCC [28, p. 392] acknowledged in its SAR that, ‘*[i]nputs to IAMs reflect the world as we know it or as we might expect it to evolve…*’. By virtue of this acknowledgement from the IPCC declared some 28 years ago, if the current Panel and IAM practitioners are unwilling to open publish scenario input data, then the modeller’s eye-view cannot be assessed in terms of its optical accuracy as to the present state of the world, nor the acuity of futures envisaged. Moreover, and importantly from a policymaker’s perspective, scenario transparency also safeguards a modelled scenario from covertly becoming a subversive device for vested interests [30] (see, for example, the American Petroleum Institute’s commissioning of ‘independent’ economic modelling for the purpose of weakening and delaying climate policy as detailed in Franta [61]^4^). The prevention of vested interests’ infiltration aids in maintaining the integrity of the science–society contract by averting the blurring of the science-policy boundary. Hence, with the IPCC operating as a boundary facilitator, the Panel’s use of contract-inconsistent, non-transparent IAM scenarios for ‘policy relevant’ guidance has further blurred that boundary.

Questions must be asked as to the presence of political bias in allegedly scientific global models [62]. Exploring the origins and consequences of different social and institutional assumptions embedded in policy modelling precludes the denial of the interpenetration of science and politics in policy analysis [62]. It is the entanglement of politics with science that threatens the integrity of and undermines the public’s trust in science [63]. Consequently, IAM practitioners have a responsibility to ensure all ‘*values and beliefs*’ are transparent in their products, despite the verity that, ‘…*even apparent transparency may hide values*’ [47, p. 46]. Such a responsibility equally applies to the IPCC with the Panel’s predominant reliance upon IAM products, especially owing to the dynamics associated with the ‘demand-pull’ science-policy boundary mode. By reason of this fact, climate change mitigation policy guidance derived from non-transparent IAMs, as discussed herein before, violates the IPCC’s professed remit of merely reporting on the state of scientific knowledge for the benefit of all policymakers from both the global North and the global South, whose end policies should be for the shared benefit of people and planet.

Low-carbon transition research is certainly not the preserve of IAMs. Low-carbon transition research, which is ultimately concerned with socio-environmental management, should not and must not be dominated by economists or worse still quasi-economists misrepresenting dated economic orthodoxes, many of whom elevate economic considerations above social and/or environmental considerations.^5^ With IAMs being dominated by neoclassical economic ideology [29], such models intrinsically fail to adhere satisfactorily to the three underpinning tenets of sustainability. This is owing to the models’ *bien pensant* state of reducing non-economic phenomena to a quantifiable economic measure. Conventional economics that are at the root of existential crises must be discredited scientifically, delegitimized academically and rejected socially [65].

## 4. Conclusion

If the IPCC was to earnestly assure the transformation of IAM clarity from its present state of a black-box to that of a glass-box, then its proclaimed mantra of ‘*neutral, policy relevant but not policy prescriptive*’ could be received with high confidence [30]. Until then, the IPCC endangers its objectivity, its integrity and its scientific standing in society owing to the Panel’s non-compliance with the published Principles Governing IPCC Work as to expected transparency standards. Adopting a chronological marker of Risbey *et al*. [12] as an epoch to this dialectical discourse indicates that the IPCC has had a quarter of a century to resolve this issue of transparency as it relates to IAMs. The Panel must immediately rectify this basic though critical issue and to fulfil that espoused in Skea *et al*. [10] if it is to remain compliant with the Principles Governing IPCC Work. Research integrity is reliant upon moving from ‘*talk to walk*’ [66].

If we are to overcome policy resistance, it will require a steadfast commitment to the highest standards, the sedulous application of the scientific method, the scrutiny skills to expose our hidden assumptions and biases and the curiosity to continually pose ‘why’ questions [43, p. 527]. With the IPCC’s continued exploitation of opaque global mitigation scenarios generated by IAMs, biases are concealed, assumptions remain hidden and curiosity is curtailed, which, in totality, thwarts stakeholders from asking those ‘why’ questions. The prevailing practice of closed integrated assessment modelling prevents the production of socially robust knowledge. Instead, IAMs produce unreliable knowledge; in other words, science that does not ‘work’^6^, which is then presented by the IPCC. The production of socially robust knowledge recognizes a need for a new science–society contract, one which champions openness and transparency while demanding the recurrent legitimization of scientific authority [49]. This is heralded by science’s departure from the ivory tower and its entrance into the agora [49]. Significantly, with societal participation in the production of socially robust knowledge from inception, such knowledge is less likely to be contested [49]. If the IPCC is to maintain its scientific authority, the Panel must evolve in response to shifting social and political mores [68].

Government inaction on rapid greenhouse gas emissions reduction can be ascribed in part to the sempiternal time-buying mitigation scenarios^7^ afforded by Delphian IAMs. These non-transparent IAM scenarios have been influenced, either directly and/or indirectly, by government, industry and financial institutions via demand–pull interactions across the science-policy boundary and promoted by the IPCC.^8^ Such a situation represents the epitome of the political cart pulling the scientific horse. Climate change policy derived from non-transparent IAMs in response to the dominant ‘demand-pull’ science-policy boundary mode has marked a shift in power. Such a power shift has, in turn, redefined the once distinctly separate roles of those vital actors who inhabit either side of the customary science-policy boundary. In the case of the 1.5°C target inception, it has been posited that an idiosyncrasy of politico-science codependency exists in that political objectives require scientific backing for legitimatization, and consequently, research must modulate in accordance with political discussions for it to remain policy-relevant [16]. This form of political calibration challenges the very notion of the political neutrality of IAMs [70] and runs the ruinous risk of policy-based evidence-making on account of climate advisers failing to maintain their integrity [71].

The subversion of particular modelling programmes by powerful industrial interests has had the effect of capturing regulatory institutions, eroding ostensible academic independence and distorting public discourse [72]. Lamentably, the complexion of routine policy modelling does create *‘fertile ground for serious deliberate manipulation*’ [72, p. 105]. Modellers must be vigilant to the explicit and implicit vectors employed by vested interests that currently colour their research activities [73]. It follows that as a principal boundary facilitator, it is critical to the credibility of the IPCC that its interface practices are perceived, in addition to being policy-relevant but not policy-prescriptive, to be science-relevant but not science-prescriptive [74]. The IPCC must attenuate the risk of vested interests influencing knowledge production processes [75]. With modelling having been found to be political, the application of similar standards to other political processes should be applied [73], particularly when the sense of authority that is projected by the seeming rigour and technical complexity of IAMs is baseless [53]. Censorship for political purposes is a regrettable element of the IPCC process [76]. The science–society contract should recognize the politics of environmental knowledge [77]. By contesting the IPCC’s claim to policy neutrality, the complexities, subjectivities and value judgements that are intrinsic to the production of scientific assessments are exposed [78]. An avoidance of accountability is only afforded by the turbidity of process where transparency is eschewed. Secrecy does not sit well with science [21].

Box & Draper [79, p. 424] aphorized—‘*…all models are wrong, but some are useful*’. However, in the case of the preceding critique, if the IPCC continues to fail in moving from talk to walk and elects to prolong the obfuscation of IAM and/or scenario transparency thereby preventing verification by all enquiring stakeholders, then perhaps a conditional and logical extension to the aphorism is required, in that*—all IAMs are wrong, and none are useful*. In light of the above discussion and confronted with the irrefutable reality of the unabating global growth in carbon dioxide emissions, most regrettably, I can only concur with Glavovic *et al*.’s statement [2, p. 1]: *The science-society contract is broken*.^9^

## Data Availability

This article has no additional data.

## Footnotes

^1^Concerningly, this glaring omission had been previously made known to the IPCC WGIII, with it having been documented in my submitted, accepted and publicly available Expert Review comments for Annex III of the Second Order Draft.

^2^Paragraph 12 states—‘In the case of author and review teams, bias can and should be managed through the selection of a balance of perspectives….In selecting these individuals, care must be taken to ensure that biases can be balanced where they exist’. Paragraph 2 states—‘The role of the IPCC demands that it pay special attention to issues of independence and bias in order to maintain the integrity of, and public confidence in, its products and processes’.

^3^Indeed, Skea acknowledges the utmost importance of scenario transparency when he lent his ‘*external voice*’ to the Shell Sustainability Report by publicly exalting the fossil fuel major’s ‘*unequalled degree of transparency*’ in ‘*publishing detailed assumptions and model outputs*’, as conveyed by Skea [35, p. 39].

^4^It is perhaps significant to note that the Stanford Energy Modelling Forum, a member of the IAMC, has in recent forums included financial contributor organizations, such as the American Petroleum Institute, Aramco Services Company, BP, Chevron, ExxonMobil and Schlumberger—https://emf.stanford.edu/industry-affiliates (last accessed 7 April 2023).

^5^Revealingly, and seemingly disregarding the role of the discount rate on mitigation deference, Keywan Riahi, Director of the Energy, Climate and Environment programme at the International Institute for Applied Systems Analysis (IIASA), a member of the IAMC and IPCC WGIII AR6 Chapter 3 co-ordinating lead author, in an interview with The Grist admitted that, ‘*The quicker we reduce emissions, the higher the cost will be because we have a lot of long-lived fossil-based infrastructure which would need to be prematurely phased out if we really try to accelerate and achieve zero emissions early*’ [64].

^6^Ziman [67], as cited in Gibbons [49], argues that science as a form of ‘reliable knowledge’ becomes established by the replicability of research statements and the formation of a consensus within the relevant peer group. This reliability is accepted as it is an expression of knowledge that ‘works’.

^7^For example, McLaren & Markusson [52] assert that the burgeoning interest in modelling cabon capture and storage (CCS) technology in IAM scenarios, initially applied to fossil fuels and then extended to bioenergy (i.e. BECCS), has had the compounding effect of decelerating the transition. These scenarios function to wickedly sustain the incautious narrative of a perpetually open window of opportunity, which, to date, has excused government inaction.

^8^On the authority of the Assistant Undersecretary of State Bill Nitze, as personally communicated to Michael Oppenheimer, the US government viewed the IPCC’s creation as a means of controlling the policy agenda [69].

^9^Albeit for reasons as discussed, other than those posited by Glavovic *et al*. [2].
